# Physicochemical Characteristics of Bone Substitutes Used in Oral Surgery in Comparison to Autogenous Bone

**DOI:** 10.1155/2014/320790

**Published:** 2014-07-08

**Authors:** Antoine Berberi, Antoine Samarani, Nabih Nader, Ziad Noujeim, Maroun Dagher, Wasfi Kanj, Rita Mearawi, Ziad Salemeh, Bassam Badran

**Affiliations:** ^1^School of Dentistry, Lebanese University, P.O. Box 4, Hadath, Lebanon; ^2^Ecole Doctorale, PRASE, Lebanese University, P.O. Box 4, Hadath, Lebanon; ^3^Faculty of Dental Medicine, Saint Joseph University, P.O. Box 17-5208, Beirut, Lebanon

## Abstract

Bone substitutes used in oral surgery include allografts, xenografts, and synthetic materials that are frequently used to compensate bone loss or to reinforce repaired bone, but little is currently known about their physicochemical characteristics. The aim of this study was to evaluate a number of physical and chemical properties in a variety of granulated mineral-based biomaterials used in dentistry and to compare them with those of autogenous bone. Autogenous bone and eight commercial biomaterials of human, bovine, and synthetic origins were studied by high-resolution X-ray diffraction, atomic absorption spectrometry, and laser diffraction to determine their chemical composition, calcium release concentration, crystallinity, and granulation size. The highest calcium release concentration was 24. 94 mg/g for Puros and the lowest one was 2.83 mg/g for Ingenios *β*-TCP compared to 20.15 mg/g for natural bone. The range of particles sizes, in terms of median size D50, varied between 1.32 *μ*m for BioOss and 902.41 *μ*m for OsteoSponge, compared to 282.1 *μ*m for natural bone. All samples displayed a similar hexagonal shape as bone, except Ingenios *β*-TCP, Macrobone, and OsteoSponge, which showed rhomboid and triclinic shapes, respectively. Commercial bone substitutes significantly differ in terms of calcium concentration, particle size, and crystallinity, which may affect their *in vivo* performance.

## 1. Introduction

Bone is a living tissue that serves for structural support and calcium metabolism. Bone matrix is organic and consists of a network of collagen protein fibers impregnated with mineral salts (85% of calcium phosphate, 10% of calcium carbonate, and 5% of calcium fluoride and magnesium fluoride). The mineral compartment of bone is predominantly present in the form of calcium hydroxyapatites (Ca_10_[PO_4_]_6_[OH]_2_). Bone tissue also contains negligible quantities of noncollagen proteins, including the family of bone morphogenetic proteins (BMPs) [1].

Calcium (Ca) plays an important role in osteoconductivity and may enhance bone tissue integration by entrapping and concentrating circulating bone growth factors (BMPs) and osteoprogenitor cells, thus imparting osteoinductive properties to calcium-based bone graft materials [2, 3].

Autogenous bone is osteogenic (cells within a donor graft synthesize new bone at implantation sites), osteoinductive (new bone is formed by active recruitment of host mesenchymal stem cells from surrounding tissue, which differentiate into bone-forming osteoblasts), osteoconductive (vascularization and new bone formation into the transplant), and highly biocompatible [3, 4]. These characteristics should be present in an ideal substitute and all bone grafting materials can be classified according to these characteristics [5].

Bone substitutes (BS) are frequently used in oral and maxillofacial surgery, periodontics, and orthopedics. They include inorganic or organic, natural or synthetic materials to compensate for bone loss or to reinforce new bone ingrowth into defect sites [6–17]. This second option is, in fact, the role played by calcium phosphate and constitutes materials that show the closet similarity to the mineral component of bone [14]. The greatest success in bone grafting has been achieved with autogenous bone (gold standard), which fulfills all essential physicochemical and biological properties needed in a bone graft material, despite its inherent limitations in availability and postoperative pain at donor sites [6, 8, 10, 11, 13, 18–20].

Numerous BS biomaterials have been successfully used, such as allografts (human), xenografts (porcine, equine, or bovine, and synthetic calcium-based materials (calcium phosphates [*β*-tricalcium phosphate/*β*-TCP, hydroxyapatite/HA], bioactive glasses), calcium sulfate, calcium hydroxide), and a combination of these with or without the use of membrane and screws [6–23].

Allografts do not have the drawbacks of autografts but are less successful in clinical practice. They also display several other disadvantages: risk of disease transmission or infection, difficulties in obtaining and processing, possible rapid resorption, [8–10, 24], and partial loss of mechanical strength after sterilization [25, 26]. Xenogenic bone substitutes of porcine, bovine, or, more recently, equine origin are used because of their chemical and structural composition similarity when compared to human bone [27]. They represent an unlimited supply of available material and may reduce morbidity by eliminating the donor site [5, 10, 22, 23]. Heat or other treatments are used to deproteinate bone particles and eliminate immunogenicity risks [25, 28]. Synthetic calcium phosphate ceramics with their excellent biocompatibility are common alternatives to autogenous bone [15].

Ideally, a BS should have specific biological and clinical particularities. Biologically, it should mediate recruitment of mesenchymal cells derived from host site and have bioactive effects on ossification (osteoinduction). Furthermore, it must be osteoconductive, providing three-dimensional scaffolds for the ingrowth of vessels and osteoprogenitor cells. Finally, it should be resorbable. Clinically, a BS should be easy to use, cost effective, and with adequate density to allow easy radiographic recognition during the entire healing process [27, 29]. This feature is particularly important to radiographically follow the rate of resorption/substitution [30, 31].

Regarding material structure, particle size affects not only contact area but also the packing characteristics of the materials, which ultimately determines the macroporosity of a particulate graft [32, 33]. It is also known that pore size exerts a major influence over the interaction of osteogenic cells with the biomaterial surface [34, 35]. Biological integration requires pores that are greater than 100–150 mm in diameter to provide a blood supply to the tissues [27]. A BS should gradually degrade with time until it is completely replaced with vital new bone tissue. Moreover, a material's resorption rate should match the formation rate of the new bone tissue [29]. Biomaterial degradation that occurs too rapidly can exert a negative effect on bone regeneration processes, [24] and the presence of residual BS graft particles after bone healing may lead to composite tissue repair rather than to bone tissue regeneration [27].

The aim of this study was to evaluate some of physical and chemical properties in a variety of commercially available granulated mineral-based biomaterials that are frequently used for dental applications as bone substitutes and to compare them with autogenous bone.

## 2. Materials and Methods

This study evaluated the physicochemical characteristics of the eight commercially available bone substitutes of human, bovine, and synthetic origins. Each material was used in its lowest available particle size range, and all samples were obtained directly from their manufacturers in sealed vials and evaluated without alteration.

- DynaBlast (Keystone Dental, Inc., Burlington, MA) is a combination of mineralized and demineralized allogenic bone that is mixed with a proprietary poloxamer reverse-phase resorbable medium and processed into a paste or puttylike form [28, 36].
- Puros bone allograft (Zimmer Dental, Inc., Carlsbad, CA) is an allogenic graft material treated by a proprietary process (Tutoplast, RTI Biologics, Inc., Alachua, FL) designed to inactivate pathogens and remove fat, cells, and antigens, while preserving the minerals and collagen matrix of the native bone tissue. After processing, the material is preserved by solvent dehydration, which can also help to ensure pathogen inactivation [13]. It is available in cortical, cancellous, and a cortical-cancellous mix in particle sizes ranging from 0.25 to 2 mm [26, 37].
- OsteoSponge allograft (Bacterin International, Inc., Belgrade, MT) consists of 100% of demineralized human cancellous bone, with no additional carrier materials. It is prepared using undisclosed methods that reportedly preserve native growth factors [25]. The granule size varies from 1 to 4 mm.
- BioOss (Geistlich Pharma AG, Wolhusen, Switzerland) xenogenic spongiosa granules are reported to be a natural bone mineral derived from bovine bone which contain carbonate apatite. Granules are rendered nonorganic through a proprietary extraction process that involves treatment with strong alkalis and organic solvents under high-temperature processing up to 300°C, which allegedly renders the substrate antigenic and protein-free [37]. The material was used in granules of 0.25–1 mm.
- Cerabone (AAP Biomaterials GmbH, Berlin, Germany) xenogenic granulate is a bovine bone material sintered at high temperature (>1200°C), which retains the inorganic part of bone (hydroxyapatite) [38]. The material used was granulate of 0.5–1.0 mm in size.
- Macrobone (Euroteknika Groupe, Sallanches, France) is a high-porosity (90%), synthetic bone substitute made of pure *β*-TCP that is completely and rapidly resorbable [39]. Particle size varies between 0.15 mm and 2 mm.
- IngeniOs *β*-TCP (Zimmer Dental, Inc.) is a bioactive material made of silicated *β*-TCP of non-biologic origin. The structure is a porous biocompatible synthetic scaffold of ceramic material [40]. The size of the particles is 0.25–1 mm.
- IngeniOs HA (Zimmer Dental, Inc.) is a synthetic spongious bone substitute. The structure is a porous scaffold that resembles cancellous bone. Particles are biocompatible and made of 100% hydroxyapatite ceramic with a putty phase of ≥95%, and granules range 1-2 mm in size [41].
- Autogenous bone samples were collected during mandibular third-molar surgery, rinsed with ethanol, dried in vacuum at room temperature, ground in an agate mortar, and sterilized by gamma irradiation [42, 43].

*Atomic Absorption Spectroscopy (AAS)* (WFX-210, RayLeigh, BRAIC, China) was used to determine the concentration of calcium ions in the bone substitutes by quantifying the release of calcium and phosphorous from the graft material in demineralized water. For this aim, standards for calcium and phosphorous within the range between 0.5 and 10 *μ*g/L were prepared, and 0.4 mg of each biomaterial (all nine samples) was immersed in 100 mL of 0.9% NaCl and the pH was adjusted at 7 by using hydrochloric acid (0.1 N). The variation of Ca concentration was determined at D_0_ (day 0), D_2_ (day two), and each week after, until the sixth week. The concentration was calculated based on the Beer-Lambert law [44].

*LASER Diffraction (LD)* was used to determine particle size by evaluating the distribution of the granules using a laser scattering particle size analyzer (Patrica LA-950 V2 Horiba Instruments, Japan). The measurement method relied on the Mie scattering theory [45]. Using an ultrasonic probe with measuring time of 20 s at a frequency of 20 kHz, the unit's measuring range varied between 0.01 and 3.00 *μ*m. The devise was equipped with an optical system of two light sources, a laser diode of approximately 1.6 mW with *λ* = 650 nm, and a 405 nm light emitting diode of approximately 0.3 mW. Large particles scatter light at small angles relative to the laser beam and small particles scatter light at large angles. The particle size is reported as a volume equivalent sphere diameter [43, 46]. Samples were well mixed and homogenized in their powder state prior to their analysis. Average particle size and distribution were calculated for all nine biomaterials and autogenous bone.

*X-ray Diffraction (XRD)* (D8 Advance, Bruker Corporation, Billerica, MA) was used to identify phase and composition features and qualitatively evaluate the crystallinity of all study materials. Homogenized powder samples (1-2 g) were compressed in polyvinyl chloride lenses (diameter 2.5 cm, thickness 2 mm) and measured using a diffract meter (copper anticathode *λ*K*α* = 0.154060 nm). A range of 2*θ* between *x*° and *y*° was chosen to obtain maximum information about crystal phases. Collected diffract grams were analyzed by software EVA (EVA, Bruker Corporation) based on powder diffraction files provided by the International Center for Diffraction Data (Newtown Square, PA). Crystallite size analysis was calculated using the peak broadening of XRD reflection that is used to estimate the crystallite size in an orthogonal direction to the crystal plane according to the following formula:

(1) $$\begin{array}{c} X_{s}=\frac{0.9\lambda }{\left(\text{FWHM}\times \cos\;\theta \right)}, \end{array}$$

where *X*_*s*_ is the crystallite size in nanometer, *λ* is the wavelength of X-ray beam in nanometer (*λ* = 0.15406 nm in our case), and FWHM is the full width at half maximum for the diffraction angle at 2*θ* = 25.9° that was selected according to (002) Miller's plane family [47].

## 3. Results

AAS results of calcium concentration over the observation period are summarized in Table 1. Cerabone showed less calcium release than BioOss. In the synthetic xenograft category, Macrobone displayed a high calcium release concentration (17.30 mg/g), compared to IngeniOs HA (2.92 mg/g) and IngeniOs *β*-TCP (2.83 mg/g). In the allograft group, OsteoSponge revealed the lowest calcium release concentration (4.05 mg/g). The calcium concentration of Puros (24.94 mg/g) was comparable to autogenous bone (20.15 mg/g).

The particles median size *D*_50_ (in volume percentages), the particle size range expressed by the 10% and 90% percentiles (*D*_10_ and *D*_90_), and the particles size ranges reported by the manufacturers as determined by the LD measurements are all presented in Table 2.

Results showed that BioOss had the lowest median particle size (1.32 *μ*m) followed by Ingenios *β*-TCP (6.72 *μ*m), while OsteoSponge had the highest one (902.41 *μ*m).

The median size of Macrobone (262.37 *μ*m) was close to autogenous bone (282.1 *μ*m). The narrowest size distribution was observed with BioOss (0.26–8.92 *μ*m), followed by Ingenios *β*-TCP (3.90–15.18 *μ*m). The widest size distribution was observed with OsteoSponge (174.62–2301.84 *μ*m) followed by DynaBlast (39.24–1754.62 *μ*m).

**Figure 1:**
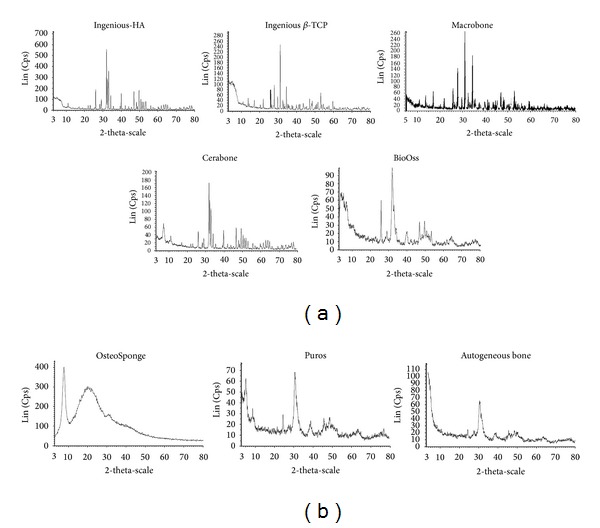
X-ray diffraction data for all investigated samples. (a) Ingenios HA, Ingenios *β*-TCP, Marrowbone, Cerabone, and BioOss have well defined peaks, which reflects their well-crystallized components. (b) OsteoSponge, Puros, and autogenous bone have noisy diffractograms revealing less crystallinity.

X-ray diffractograms for all bone substitutes are shown in Figure 1. They represent the intensity of X-ray (cps) as a function of the diffraction angles (2 theta, *θ*).

Results of the XRD experiments that are indicative for the chemical composition of the BS are shown in Table 3 except for DynaBlast, as the puttylike material was not granular in form. All study materials showed small amounts of impurities. These materials diffract more and less the X-ray, which means diverse degrees of crystallinity, as indicated in the different peaks widths.

The common crystal phase was calcium phosphate silicate hydroxide (Ca_5_(PO_4_)_2.85_(SiO_4_)_0.15_(OH)) in BioOss, Ingenios HA, Puros, OsteoSponge, and autogenous bone. Macrobone was composed from calcium phosphate (Ca_3_(PO_4_)_2_). Cerabone and Ingenios *β*-TCP were composed, respectively, of calcium gadolinium oxide phosphate (Ca_8_Gd_2_(PO_4_)6O_2_) and sodium calcium iron phosphate (Na_2_Ca_19_Fe_0.667_(PO_4_)_14_) as the main crystal phases. Except for Ingenios *β*-TCP, Macrobone, and Osteosponge, all samples were crystallized at different levels of crystallinity in hexagonal systems. The *a*/*c* or *b*/*c* ratios (9.42/6.89) indicated a flat structure parallel to *A*_6_ axis. Such geometry may enhance the settlement properties of these particles. For Ingenios *β*-TCP and Macrobone, *a* and *b* crystal dimensions in the rhombohedra system were relatively too close to *a* and *b* dimensions of the other bones crystallized in hexagonal system. Nevertheless, *c* length (37.3 Å) in Ingenios *β*-TCP and Macrobone was 5.4 times greater than *c* (6.89 Å) length in the other bones. Hence, settlement may be oriented preferably orthogonal to *A*_3_ axis. In Ingenios *β*-TCP and Macrobone, crystal size was greater than that of the other bones. OsteoSponge was the only sample crystallized in the triclinic system.

## 4. Discussion

The higher the calcium concentration in a biomaterial, the more prone it will be to degradation [3, 42, 43]. The acidic buffer, to some extent, mimics the acidic environment during osteoclastic activity or bone resorption [3, 42, 43]. In our study, different biomaterials had different calcium releasing characteristics. This could be explained by the fact that the speed of BS biodegradability* in vivo* or* in vitro* depends on the material's composition, particle size, crystallinity, porosity, and preparation [3, 38, 42, 43, 48].

From the particle size data, it can be concluded that, in general, size ranges measured for tested materials were different from those reported by manufacturers who do not specify the technique used in the crystalline material's characterization and could explain the noticed differences [49–54]. However, it should be kept in mind that the granules under analysis differed not only in their size but also in their physicochemical properties.

**Figure 2:**
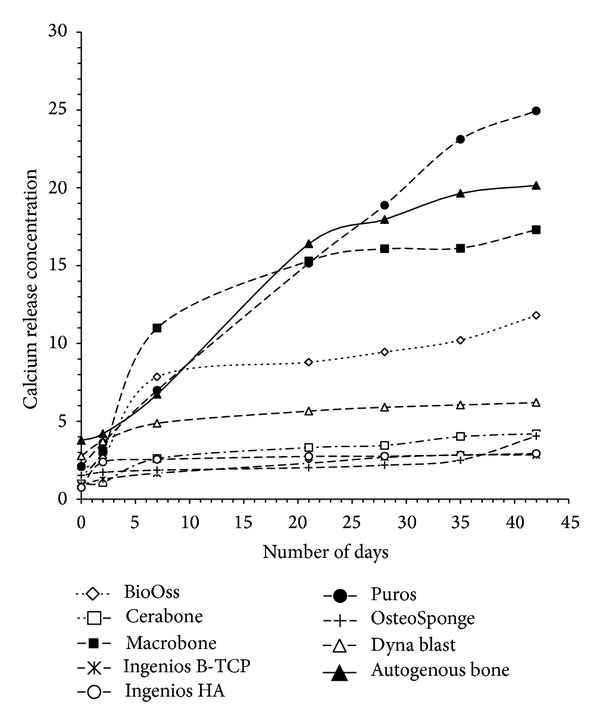
AAS results from calcium concentration over the observation period of all tested bone substitutes. *Y*-axes represent calcium release in mg/g and *X* axes represent day's number.

The influence of properties and characteristics of BS on biological response cannot be easily predicted as the published studies involve different types of BS in different particle size ranges. Regarding the ranges of particle size that were tested in the present investigation, there was no relation between the sizes of particles and calcium concentration with the time (Figure 2).

X-ray diffractograms give a clear idea about the crystallinity of the analyzed materials and their crystal phases. HA, Ingenios *β*-TCP, Macrobone, Cerabone, and BioOss have well-defined peaks which reflects their well-crystallized components; OsteoSponge, Puros, and autogenous bone have noisy diffractograms revealing the presence of amorphous structure or at least noncrystallized faces of the materials.

All bone substitutes show a typical and most intense diffraction at 2*θ* of 32° since phosphate is the common component in all used materials. Crystal phases were identified using the powder diffraction files, provided by the Interactive Center for Diffraction Data.

XRD diffractograms of various materials, including human bone, were quite similar to common crystal phase calcium phosphate silicate hydroxide (Ca_5_(PO_4_)_2.85_(SiO_4_)_0.15_(OH)) except for Cerabone, which showed the presence of gadolinium in its composition, and Ingenios *β*-TCP, which showed the presence of iron. However, silicates, when they are present, are not major components of the crystal phases, since their stoichiometry compared to phosphate (2.85) were considerably negligible (0.15). Iron and sodium are also negligible compared to calcium in Ingenios *β*-TCP. Along with calcium, they help to compensate the negative charges of phosphate. It must be highlighted that, in bone and all bone substitutes, Ca to P ratios fluctuated between 1.75 and 1.33. This could mean that calcium was the major element that compensated phosphate charges.

It is not clear why gadolinium was present in the crystal phase of Cerabone. One possible explanation could be the iron oxidized at high temperature since the product was subjected to high-temperature calcination ±1200°C [55]. Another explanation could be that it was used for its property to enhance the resistance of alloys against oxidation. It should be noted that natural gadolinium occurs in monazite mineral (rare earth phosphate) and gadolinium salt has an exceptionally high absorption of neutrons and therefore is used for shielding in radiography as a contrast agent [56].

Nevertheless, we could not assert if gadolinium in those studied samples naturally occurred or was purposely added.

XRD diffractograms showed that all samples, including natural bone, proved to have the same anisotropic crystal size (9.42 Å in *a*- and *b*-directions and 6.89 Å in *c*-direction with alpha and beta 90° and gamma 120°); Ingenios *β*-TCP and Macrobone showed different anisotropic size 10.4 Å in *a*- and *b*-directions and 37,3 Å in *c*-direction with alpha and beta 90° and gamma 120°, 6.25 Å in *a*-direction, 11.9 Å in *b*-direction, and 5.6 Å in *c*-direction with alpha 97 and beta 114° and gamma 93°, respectively. These results demonstrated that crystal shapes of the BS and autogenous bone had a similar, hexagonal shape; only Ingenios *β*-TCP and Macrobone showed a rhomboid design and OsteoSponge a triclinic shape. This structure is the poorest system in the symmetric properties [57].

Eight different bone-grafting materials were herein investigated, and the results were compared to autogenous bone. Even when similar chemical characteristics were found, significant differences were detected in terms of calcium concentrations, particle sizes, and crystallinity. Although these morphological differences greatly influence* in vivo* behavior of the biomaterial, they are often not taken into consideration when the samples' biological performance is evaluated. It is believed that results provided for biomaterials investigated will be most useful to fully understand their clinical behavior and response. Since the bone substitute of choice depends largely on the possible clinical application and its associated biological and mechanical needs, it is important not to assume that all bone substitutes will show the same pattern of performance and that the validation of a bone substitute in one clinical site may not necessarily predict its identical performance in another anatomical location. Hopefully in the future, hybrid or complex combination products that include cells, growth factors, and/or gene therapy in combination will be likely to provide oral surgeons more effective tools for bone defects reparation. In this regard, it is obvious that further studies are warranted and a new international standard for characterization, classification, and identification of implantable materials is needed.

## 5. Conclusion

Commercial bone substitutes significantly differ in terms of calcium concentration, particle size, and crystallinity from autogenous bone, which may affect their clinical applications and performance.

## Figures and Tables

**Table 1:** The calcium concentration as derived from AAS experiments by brand names and time period.

Ca (mg/g)	BioOss	Cerabone	Macrobone	Ingenios B-TCP	Ingenios HA	Puros	OsteoSponge	Dyna Blast	Autogenous bone
Day 0	1.977	0.98	1.4485	0.892	0.7485	2.104	1.521	2.768	3.77
Day 2	2.643	1.061	3.0735	1.276	2.3862	3.613	1.723	3.7132	4.2
Week 1	7.849	2.605	10.9865	1.663	2.5515	6.99	1.859	4.871	6.73
Week 3	8.79	3.308	15.301	2.306	2.7427	15.1535	2.018	5.6507	16.4
Week 4	9.451	3.445	16.081	2.682	2.7502	18.879	2.18	5.8985	17.96
Week 5	10.205	4.023	16.11	2.834	2.8282	23.11	2.493	6.0485	19.64
Week 6	11.8	4.194	17.308	2.835	2.9275	24.942	4.051	6.2	20.15

**Table 2:** Particle size parameters in volume percentage of the samples.

Sample	Median size	Size range	Size range reported
	(D_50_ *μ*m)	(D_10_–D_90_ *μ*m)	By producers (*μ*m)
Bio-Oss	1.32	0.26–8.92	250–1000
Cerabone	663.31	174.62–1337.48	500–1000
Macrobone	262.37	22.79–517.2	150–500
Ingenios B-TCP	6.72	3.90–15.18	250–1000
Ingenios HA	592.39	8.82–1337.48	1000–2000
Puros	630.47	174.62–1167.72	250–2000
OsteoSponge	902.41	152.45–2301.84	1000–4000
Dyna Blast	777.14	39.24–1754.62	Nonindicated
Autogenous bone	282.1	90.5–465.15	

**Table 3:** Chemical compositions and shapes of samples, except for Dyna Blast due to its puttylike form, as derived from X-ray diffraction.

Product	Compound name	Formula	System	*a* (Å)	*b* (Å)	*c* (Å)	Alpha (°)	Beta (°)	Gamma (°)
Bio-Oss	Calcium phosphate silicate hydroxide	Ca_5_(PO_4_)_2.85_(SiO_4_)_0.15_(OH)	Hexagonal	9.42	9.42	6.89	90	90	120
Cerabone	Calcium gadolinium oxide phosphate	Ca_8_Gd_2_(PO_4_)_6_O_2_	Hexagonal	9.39	9.39	6.89	90	90	120
Macrobone	Calcium phosphate	Ca_3_(PO_4_)_2_	Rhomboid	10.4	10.4	37.4	90	90	120
Ingenios B-TCP	Sodium calcium iron phosphate	Na_2_Ca_19_Fe_0.667_(PO_4_)_14_	Rhomboid	10.4	10.4	37.3	90	90	120
Ingenios HA	Calcium phosphate silicate hydroxide	Ca_5_(PO_4_)_2.85_(SiO_4_)_0.15_(OH)	Hexagonal	9.42	9.42	6.89	90	90	120
Puros	Calcium phosphate silicate hydroxide	Ca_5_(PO_4_)_2.85_(SiO_4_)_0.15_(OH)	Hexagonal	9.42	9.42	6.89	90	90	120
OsteoSponge	Calcium phosphate silicate hydroxide	Ca_5_(PO_4_)_2.85_(SiO_4_)_0.15_(OH)	Triclinic	6.25	11.9	5.6	97	114	93
Dyna Blast									
Autogenous Bone	Calcium phosphate silicate hydroxide	Ca_5_(PO_4_)_2.85_(SiO_4_)_0.15_(OH)	Hexagonal	9.42	9.42	6.89	90	90	120
